# Maternal plasma 25-hydroxyvitamin D concentration and birthweight, growth and bone mineral accretion of Gambian infants

**DOI:** 10.1111/j.1651-2227.2009.01352.x

**Published:** 2009-08

**Authors:** Ann Prentice, Landing MA Jarjou, Gail R Goldberg, Janet Bennett, Tim J Cole, Inez Schoenmakers

**Affiliations:** 1 MRC Human Nutrition Research, Elsie Widdowson LaboratoryCambridge, UK; 2 MRC KenebaThe Gambia; 3 MRC Centre of Epidemiology for Child Health, UCL Institute of ChildHealth, London, UK

Maternal vitamin D deficiency during pregnancy is a recognized risk factor for rickets and osteomalacia in infancy (1). The circulating plasma concentration of 25-hydroxyvitamin D (25OHD), a long-lived metabolite of vitamin D, is used to judge vitamin D status; values below 25 nmol/L are associated with an increased risk of rickets and osteomalacia (1). There is evidence that a low maternal plasma 25OHD in pregnancy may influence the growth and bone mineral accrual of the offspring during foetal life, infancy and childhood. Positive associations have been reported between maternal vitamin D status in pregnancy and birthweight, birth length, length at 1 year and bone mineral accretion at 9 years (2–6), although evidence is conflicting (7,8). These relationships have been observed at concentrations of 25OHD higher than those associated with rickets and osteomalacia, and there are calls to raise the accepted lower threshold of vitamin D sufficiency for pregnant women, most recently to 80 nmol/L (9). On a population basis, plasma 25OHD concentrations above 80 nmol/L are relatively uncommon in countries at temperate latitudes but are more common among people living in the tropics who have abundant skin sunshine exposure (10). To contribute to the debate on the definition of vitamin D sufficiency in pregnancy, we have investigated the influence of maternal plasma 25OHD concentration on foetal and infant growth in a rural area of The Gambia, West Africa (13°N). In this region, there is tropical sunshine all year, the women are farmers who work out-of-doors for much of each day, and local female dress does not restrict regular sunshine exposure to the face, neck, shoulders, arms and feet, especially during farm work and gardening.

The study was a secondary analysis of biochemical, anthropometric and bone data from a subset of 125 women and infants collected during a calcium supplementation study of blood pressure in pregnant Gambian women (International Trial Registry: ISRCTN96502494). No significant benefits for foetal and infant growth of maternal calcium supplementation were identified despite the low customary calcium intake in The Gambia (11). The protocol, methods, maternal characteristics and infant data from the detailed study have been published (11). Briefly, women from the rural villages of Keneba and Manduar, West Kiang, The Gambia were recruited at 20 weeks of pregnancy (P20) and randomized to a daily calcium supplement or a matching placebo tablet until parturition (1500 mg Ca as calcium carbonate and microcellulose-lactose, respectively; Nycomed Pharma AS, Asker, Norway). Fasting, early morning blood was collected and anthropometry performed at P20 and 36 weeks of pregnancy (P36). The mean (± SD) age, weight, height and dietary calcium intake of the women at P20 were 27.4 ± 7.5 years, 56.3 ± 6.7 kg, 1.61 ± 0.05 m and 356 ± 190 mg/day, respectively. The median parity (range) was 3 (0–10). Infant birthweight was measured within 24 h of delivery. Weight, crown-heel length and head circumference were measured at 2, 13 and 52 weeks postpartum. In addition, infant bone mineral content (BMC), bone mineral density (BMD) and bone width (BW) or bone area (BA), were measured by single photon absorptiometry of the midshaft radius (Lunar SP2, Lunar Corporation, Madison, WI, USA) and, for a subset (n = 44, 47 and 52 at 2, 13 and 52 weeks, respectively), by whole-body dual-energy X-ray absorptiometry (Lunar DPX+, software version 4.7b, Lunar Corporation).

Plasma 25OHD was measured a using radioimmunometric assay (Diasorin Ltd, Wokingham, Berks, UK), with assay performance monitored through the Vitamin D External Quality Assessment Scheme (DEQAS; Endocrine/Oncology Laboratory, Charing Cross Hospital, London, UK). The intra- and inter-assay coefficients of variation were 4% and <6%, respectively. Relationships between infant outcomes and maternal 25OHD concentration were explored using multiple linear regressions (DataDesk 6.2.1, Data Description Inc., Ithaca, NY, USA). The following potential confounders were included in the full models: maternal weight, weight gain, height, parity, supplement group, sex of infant and season. Nonsignificant variables were removed by backwards elimination to produce parsimonious models. Infant BMC was corrected for bone and body size by including infant weight, length and BW or BA, in the regression models (12). An interaction term, supplement group × 25OHD, was added to investigate potential synergistic effects of calcium supplementation and maternal vitamin D status on infant growth outcomes. Other analyses of maternal 25OHD included comparisons between P20 and P36, and also as outcomes the proportions above and below the cut-off of 80 nmol/L. A conservative level of significance of p ≤ 0.01 was adopted in recognition of the large number of separate statistical tests performed (>100); possible trends in the data with p = 0.01–0.1 were noted. The analysis was conducted on 123 mother–infant pairs; blood samples from two subjects were not available.

**Figure 1:**
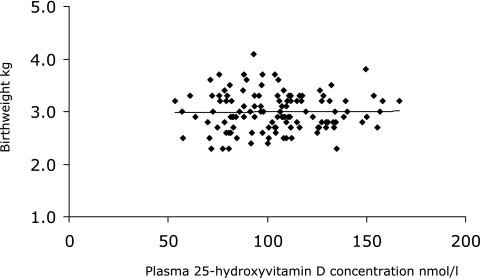
Lack of a significant relationship between infant birthweight and maternal vitamin D status at 20 weeks of pregnancy (p = 0.8). Multiple regression model included season, maternal height, weight, weight gain, supplement group and sex of the infant.

Mean ± SD 25OHD (range) was: P20 = 103 ± 25 (53–167) nmol/L; P36 = 111 ± 27 (51–189) nmol/L. No subject had a 25OHD value <50 nmol/L, 20% and 16% had 25OHD <80 nmol/L, at P20 and P36, respectively. There was a high degree of within-subject consistency in 25OHD at P20 and P36 (25OHD_P36_ = 33.2 + [0.79 ± 0.07]× 25OHD_P20_, p ≤ 0.001, *R*^2^ adjusted 51.5%, n = 121); 11% of women had 25OHD <80 nmol/L at both P20 and P36. The mean birthweight of the infants was 2.99 ± 0.36 kg. The infant anthropometric and bone measures during the first year are given in Table 1. No significant relationships or trends in the data were observed between maternal 25OHD concentration using the values at P20, P36 or the mean of the two and any of the following infant measures: birthweight, infant weight, length, head circumference, BMC, BW (or BA), BMD and size-adjusted BMC of the midshaft radius and whole body at any time postpartum. This is illustrated in Figure 1 for birthweight as a function of maternal 25OHD concentration at P20. Comparing the results for mothers with 25OHD above and below 80 nmol/L did not alter this finding.

**Table 1:** Anthropometric and bone measures of Gambian infants

Characteristic	Age (weeks) 2 (n = 125)	13 (n = 123)	52 (n = 121)
Weight (kg)	3.32 ± 0.47	5.84 ± 0.72	8.08 ± 0.97
Length (cm)	50.5 ± 1.9	59.6 ± 2.0	71.9 ± 2.8
Head circumference			
(cm)	35.5 ± 1.6	40.0 ± 1.3	44.6 ± 1.4
Weight SDS	−1.08 ± 0.86	−0.37 ± 0.98	−1.96 ± 1.15
Length SDS	−0.80 ± 0.91	−0.41 ± 0.92	−1.21 ± 1.11
Radius shaft*			
Bone mineral			
content (g/cm)	0.056 ± 0.014	0.068 ± 0.018	0.087 ± 0.022
Bone width (cm)	0.374 ± 0.066	0.470 ± 0.067	0.594 ± 0.092
Whole body†			
Bone mineral			
content (g)	50.9 ± 11.6	89.5 ± 21.4	169.6 ± 35.1
Bone area (cm^2^)	105 ± 20	177 ± 34	279 ± 50

Data are mean ± SD.

SDS, standard deviation score relative to British reference data (14).

* n = 116, 118, 96 at 2, 13 and 52 weeks, respectively.

† n = 44, 47, 52 at 2, 13 and 52 weeks, respectively.

No significant interaction between supplement group and maternal 25OHD concentration was observed for any infant variable. Trends in the data were observed in a few instances for a supplement group × 25OHD interaction among the bone measures but no consistent picture emerged and they were considered to have arisen by chance.

We conclude that there is no evidence for an influence of vitamin D status during pregnancy on infant growth and bone mineral accrual in the conditions prevailing in The Gambia. The children in this study, as is common in this region of The Gambia (13), were born small, grew well for the first months of life but experienced growth faltering during later infancy compared to Western children (11,14), as demonstrated by their weight and length SDS. The 25OHD concentrations of the women were >50 nmol/L in the second half of pregnancy, and no distinction could be drawn in infant outcomes between mothers with concentrations above or below 80 nmol/L. Thus, our study suggests that, for women with regular, adventitious UVB sunshine exposure and in situations where foetal and infant growth may be constrained by multiple factors, there would be no benefit for foetal and infant growth or bone mineral accrual in aiming to increase the vitamin D status of individual mothers during pregnancy above 50 or 80 nmol/L.
